# Reduced NKX2.1 Expression Predicts Poor Prognosis of Gastric Carcinoma

**DOI:** 10.1371/journal.pone.0114556

**Published:** 2014-12-05

**Authors:** Bai-Wei Zhao, Shan-Shan Jiang, Yong-Ming Chen, Chun-Yu Huang, Yuan-Fang Li

**Affiliations:** 1 State Key Laboratory of Oncology in Southern China and Department of Experimental Research, Sun Yat-sen University Cancer Center, Guangzhou, People's Republic of China; 2 Department of Gastric & Pancreatic Surgery, Sun Yat-sen University Cancer Center, Guangzhou, People's Republic of China; West German Cancer Center, Germany

## Abstract

Thyroid transcription factor-1 (NKX2.1/TITF-1) is a member of the thyroid tissue-specific transcription factor family that has been proven to be closely associated with many human diseases. Recently, it was reported that NKX2.1 expression is lost or reduced in some human cancers such as lung cancer and thyroid cancer. However, there was insufficient data to suggest that NKX2.1 functionality could be used as a prognostic factor. Therefore, this study aims to investigate NKX2.1 expression and its prognostic significance in primary gastric carcinoma. Then, we attempted to investigate if NKX2.1 expression was related to the clinicopathological characteristics and prognosis of gastric carcinoma (GC)patients. The expression levels of NKX2.1 were analyzed in tissue samples from 205 gastric carcinoma patients by real-time quantitative PCR (qRT-PCR), Western blotting, and immunohistochemical staining(IHC). Our qRT-PCR results showed that the expression of NKX2.1 mRNA was reduced in tumor tissue samples compared with that in matched adjacent non-tumor tissue samples (P<0.001); this finding was confirmed by Western blot analysis (P<0.001). Our immunohistochemical staining data indicated that NKX2.1 expression was significantly decreased in 87 of 205 (42.4%) gastric carcinoma cases. Kaplan-Meier survival curves revealed that the decreased expression of NKX2.1 was significantly associated with poor prognosis in gastric carcinoma patients (P<0.001). Multivariate Cox analysis identified NKX2.1 expression as an independent prognostic factor for overall survival (P = 0.005). Furthermore, the functions of Nkx2.1 were analyzed with respect to the proliferation, migration, and invasion of GC cell lines. Our data suggest that NKX2.1 may function as a tumor suppressor in primary gastric carcinoma and that its reduced expression independently predicts an unsatisfactory prognosis in gastric carcinoma patients.

## Introduction

Gastric cancer is one of the most common malignant tumor types worldwide [1] . According to global cancer statistics, gastric cancer (GC) is the fourth most common malignant tumor type and the second most common cause of cancer-related deaths (of which it accounts for 11%), with approximately 1,000,000 new cases and 800,000 deaths occurring in 2011 [2], [3]. Treatment of gastric cancer previously consisted of surgery, chemotherapy, and radiation therapy, but there were no effective targeted therapies [4] . Therefore, many researchers are investigating the molecular markers of GC to uncover new therapeutic targets [5], such as Her-2, which has been proven to be positively expressed in some GC patients and associated with disease prognosis and is being studied in the ToGA trial [6]. It has been proven that GC tumor progression is associated with a multistep process involving the activation of oncogenes and the inactivation of tumor suppressor genes [7] . Thus, we seek to better understand the molecular mechanisms of cancer progression and to develop new therapeutic tools based on these mechanisms.

Thyroid transcription factor-1 (NKX2.1/TTF-1), which was first discovered in thyroid tissue, functions as a tissue-specific upstream transcription factor. NKX2.1 mRNA has a restricted tissue distribution [8] . It is expressed at high levels in thyroid and lung tissues and at low levels in other normal tissues such as the forebrain and pituitary gland [9] . Previous studies mainly focused on the association between NKX2.1 and the differentiation of normal tissues. However, researchers recently began to investigate NKX2.1 expression in human tumor tissues and found reduced NKX2.1 expression in some types of cancer, including lung cancer, thyroid cancer and ovarian carcinomas [10]–[17], which suggested that NKX2.1 may be a putative new tumor suppressor in multiple types of human cancers. However, to the best of our knowledge, there were no previous reports concerning the expression status and prognostic value of NKX2.1 in primary gastric carcinoma.

In our study, the expression of NKX2.1 in primary gastric carcinoma was assessed using quantitative real-time PCR (qRT-PCR), western blotting and immunohistochemistry. Additionally, we identified the relationship between NKX2.1 expression and clinicopathological features, and we evaluated its prognostic value with respect to post-resection survival in gastric cancer.

## Materials and Methods

### Ethics statement

This research was approved by the Ethics Committee of the Sun Yat-sen University Cancer Center, and written informed consent was obtained from each patient involved in the study.

### Patients and Samples

Clinicopathological data from 205 gastric cancer patients who underwent surgical resection at Sun Yat-sen University Cancer Center from January 2003 to December 2006 were retrospectively analyzed. Patients who met the following eligibility criteria were included: (1) diagnosis of gastric carcinoma identified by histopathological examination; (2) surgical history that included gastrectomy and lymphadenectomy; (3) availability of complete follow-up data; (4) no preoperative treatment, such as chemotherapy and radiotherapy; (5) no history of familial malignancy or other synchronous malignancy (such as GIST, esophageal cancer, or colorectal cancer); (6) no recurrent or remnant gastric cancer; and (7) no death during the perioperative period. The tumor resection and D2 lymphadenectomy were performed by experienced surgeons, and the surgical procedures, which followed the Japanese Gastric Cancer Association (JGCA) guidelines, were similar in all patients who underwent radical resections.

Fresh gastric cancer and adjacent non-tumor tissue samples (n = 31) were obtained from 31 gastric cancer patients who underwent surgical resection at the Sun Yat-sen University Cancer Center between 2009 and 2011. After surgical resection, the fresh tissue samples were immediately immersed in RNAlater (Ambion, Inc., USA) and stored at 4°C overnight to allow thorough penetration of the tissues; the samples were then frozen at −80°C until RNA extraction. Both the tumor tissues and the adjacent non-tumor tissues, which were located more than 3 cm away from the gastric cancer, were sampled and verified by pathological examination. Paraffin-embedded samples were obtained from the 205 gastric cancer patients who underwent surgical resection at the Sun Yat-sen University Cancer Center between 2003 and 2006. Each tumor sample was assigned a histological grade based on the World Health Organization (WHO) classification criteria. All the patients were staged using the 7th edition of the International Union against Cancer (UICC) Tumor-Node-Metastasis (TNM) staging system.

### Extraction of total RNA and real-time quantitative PCR

Total RNA was extracted using TRIzol (Invitrogen, Carlsbad, California, USA) according to the manufacturer's protocol. The total RNA concentration was assessed by measuring its absorbance at 260 nm using a NanoDrop spectrophotometer (ND-1000, Thermo Scientific, USA). Reverse transcription (RT) to synthesize the first-strand of cDNA was performed with M-MLV reverse transcriptase (Promega, USA) according to the manufacturer's recommendations, using 2 µg of total RNA as a template. To evaluate the relative mRNA levels of NKX2.1 and GAPDH (used as an internal control), the resulting cDNA was subject to real-time quantitative PCR analysis using the following primers: NKX2.1 forward, 5'-GGACGGGAGCTGGGGAGAGG-3', and reverse, 5'-ATTTTCGCGGAGGGCGGTCG-3'; and GAPDH forward, 5′-CTCCTCCTGTTCGACAGTCAGC-3′, and reverse: 5′-CCCAATACGACCAAATCCGTT-3′. Gene-specific amplification was performed using an ABI 7900HT real-time PCR system (Life Technologies, Carlsbad, California, USA) with a 15-µl PCR mix containing 0.5 µl of cDNA, 7.5 µl of 2 x SYBR Green master mix (Invitrogen, Carlsbad, California, USA), and 200 nM of the appropriate oligonucleotide primers. The mix was preheated at 95°C (10 min) and then amplified at 95°C (30 sec) and 60°C (1 min) for 45 cycles. The resolution curve was measured at 95°C for 15 sec, 60°C for 15 sec and 95°C for 15 sec. The Ct (threshold cycle) value of each sample was calculated by the instrument's software (SDS 2.3), and the expression levels of NKX2.1 mRNA were normalized to that of GAPDH. The data were analyzed using the comparative threshold cycle (2-ΔCT) method.

### Western blot analysis

The frozen tissue samples from patients with gastric cancer, including the tumor and normal tissue, were homogenized in RIPA lysis buffer, then the lysates were cleared by centrifugation (12,000 rpm) at 4°C for 15 min. Approximately 40 µg of each protein sample was resolved on a 12% SDS-PAGE gel and transferred to PVDF membranes. After blocking for 60 min with 5% non-fat milk, the membranes were incubated overnight at 4°C with a primary polyclonal antibody against NKX2.1 (Abcam, USA, diluted 1∶1000). The membranes were then washed three times with TBST for 10 min each and probed with an HRP-conjugated secondary antibody (Immunology Consultants Laboratory, USA, diluted 1∶2000) for 60 min at room temperature. The membranes were then washed three times with TBST and developed using an enhanced chemiluminescence system (ECL, Pierce).

### Immunohistochemistry analysis

The tissue sections were deparaffinized with dimethylbenzene and rehydrated in 100%, 95%, 90%, 80% and 75% ethanol. After three washes in phosphate-buffered saline (PBS), the slides were boiled in antigen retrieval buffer containing 0.01 M sodium citrate-hydrochloric acid (pH = 6.0) for 15 min in a microwave oven. After rinsing with PBS, the tissue sections were incubated with primary antibody. The slides were then rinsed in 3% peroxidase quenching solution (Invitrogen) to block endogenous peroxidase. The sections were incubated with a mouse monoclonal antibody against NKX2.1 (Abcam, USA, at a 1∶500 dilution) at 4°C overnight, followed by incubation with horseradish peroxidase (HRP) (ChemMateTM DAKO EnVisionTM Detection Kit) at room temperature for 30 min. After washing in PBS, the antigen-antibody complex were visualized using 3, 3'-diaminobenzidine (DAB), and all the slides were counterstained with hematoxylin. Our negative controls were adjacent sections processed as described above but incubated overnight at 4°C in blocking solution without the primary antibody.

### Semi-quantitative method

The total NKX2.1 immunostaining score was defined as the product of scores allocated based on the percentage of positively stained tumor cells and on the staining intensity. Both the percentage of positive cells and the staining intensity were evaluated under double-blind conditions. Briefly, the percentage of positive staining was scored as 0 (0–9%, negative), 1 (10%–25%, sporadic), 2 (26%–50%, focal) or 3 (51%–100%, diffuse). The staining intensity was scored as 0 (no staining), 1 (weak staining), 2 (moderate staining) or 3 (dark staining). The total immunostaining score was calculated as the score given based on the percentage of positively stained cells multiplied by the staining intensity score, and it ranged from 0 to 9. The expression level of NKX2.1 was defined as follows: “−” (negative, score 0), “+” (weakly positive, score 1–3), “++” (positive, score 4–6), and “+++” (strongly positive, score 7–9). Based on their NKX2.1 expression levels, the gastric cancer patients were divided into two groups: the low NKX2.1 expression group (NKX2.1-) and the high NKX2.1 expression group (NKX2.1+, NKX2.1++ or NKX2.1+++).

### Cell culture and cellular growth rate

The human GC cell lines, AGS, MKN- 45, SGC-7901, HGC-27, MPC- 803, and the normal liver cell line GES were obtained from the Committee of Type Culture Collection of the Chinese Academy of Sciences (Shanghai, China). All cells were cultured in RPMI 1640 supplemented with 10% fetal bovine serum (FBS) in 5% CO2 at 37°C.

### RNA oligonucleotides

The siRNAs for the NKX2.1 knockouts were synthesized by GenePharma (Shanghai, China). The siRNA sequences were as follows: siNKX2.1, sense = 5′- GACGCUUCAAGCAACAGAATT-3′ and antisense = 5′- UUCUGUUGCUUGAAGCGUCTT-3′; and negative control (NC), sense = 5′- UUCUCCGAACGUGUCACGUTT-3′and antisense = 5′- ACGUGACACGUUCGGAGAATT-3′.

### Recombinant adenovirus and tumor cell infection

The AD-NKX2.1 and AD-GFP (as a negative control) were bought from HanHeng Biology Company in Shanghai Province, China. The recombinant adenoviruse was stored at −80°C for use. The SGC-7901 cells were cultured in 6-well plates and infected with adenovirus (Ad-NKX2.1 and Ad-GFP) at a multiplicity of infection (MOI) of 20. The efficiency of infection was estimated by western-blot

### Cell proliferation assay

Cellular growth curves were plotted using cellular viability values measured by the MTS method (Cell Titer 96 Aqueous One Solution Cell Proliferation Assay solution; Promega). GC cells transfected with gal-siRNA, NC , galectin-3-overexpression were seeded at 1000/cells per well in 96-well flat-bottomed microtiter plates in a final volume of 200 µL of culture medium per well. Cells were incubated in a humidified atmosphere (37°C and 5% CO2) for 24, 48, 72, 96, 120, 144, 168 hours after transfection. MTS assays were used to measure cell proliferation, with 5 replicates at each time point. Statistical analyses were carried out using the two-tailed unpaired Student's *t*-test.

### Cell migration assays

For transwell migration assays, 5.0 × 10^4^ gastric cancer cells in 200 µl of serum-free DMEM were added to cell culture inserts with an 8-µm microporous filter without an extracellular matrix coating (Becton Dickinson Labware, Bedford, MA). Fivehundred microliters of DMEM containing 10% FBS was added to the bottom chamber. After 24 h of incubation, the cells on the lower surface of the filter were fixed, stained and examined microscopically. The stained cells were counted under an inverted microscope (10 fields per membrane). Each experiment was performed in triplicate. Statistical analyses were carried out using the two-tailed unpaired Student's *t*-test.

### Matrigel invasion assay

The Matrigel invasion assay was performed in a chamber system consisting polycarbonate membrane inserts with 8-mm pores (Corning, USA) placed in 24-well cell culture insert companion plates. The inserts were coated with a thin layer of 0.5 mg/ml Matrigel Basement Membrane Matrix (BD Biosciences, Bedford, MA). The invasion assay was conducted at 48 hours after the cancer cells were infected with Ad-control/Ad-NKX2.1/siNKX2.1. The cells (1×10^5^ in 200 µl of growth medium without FBS) were placed in the upper chamber, and 0.5 ml of growth medium containing 10% FBS was placed in the lower chamber. The cells were incubated at 37°C and allowed to invade through the Matrigel layer for 48 hours. After incubation, the insert membranes were fixed with 75% methanol for 30 hours. The cells on the upper surface were removed with cotton-tipped swabs, and the invading cells on the lower surface were stained with 0.5% crystal violet containing 20% methanol for 60 minutes. The stained cells were counted under an inverted microscope (10 fields per membrane). Each experiment was performed in triplicate. Statistical analyses were carried out using the two-tailed unpaired Student's *t*-test

### Statistical analysis

Differences in mRNA and protein expression between the tumor samples and the paired adjacent non-tumor tissue samples were evaluated with a paired-samples t-test. The χ2 test was used to analyze the relationships between NKX2.1 expression and various clinicopathological parameters. Survival curves were calculated using the Kaplan–Meier method and compared by the log-rank test. The Cox proportional hazards regression model was used for univariate and multivariate analyses to study the effects of the clinicopathological variables and NKX2.1 expression on survival. The results were expressed as mean ± SD and analyzed using the Students't-test. Differences were considered significant at p < 0.05.

## Results

### NKX2.1 mRNA expression analyzed with qRT-PCR

**Figure 1:**
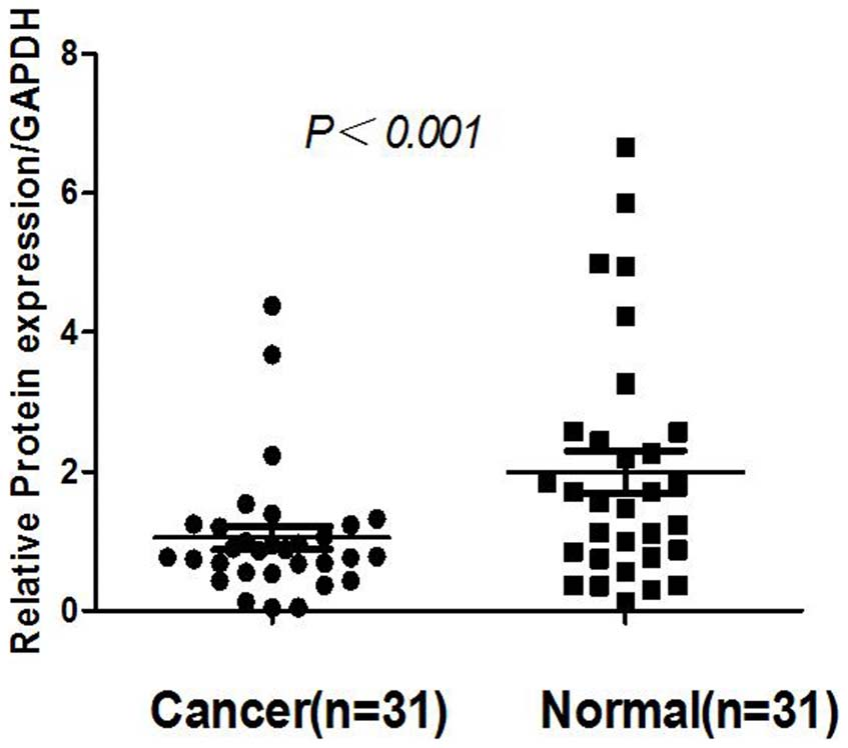
Real-time quantitative PCR analysis of NKX2.1 mRNA expression in gastric cancer patients. The relative mRNA expression of NKX2.1 was lower in 23 gastric tumor tissues than in adjacent non-tumor tissues (p<0.001).

Transcription of the NKX2.1 gene was assessed by qRT-PCR analysis of 31 pairs of resected specimens (tumor tissue samples and matched adjacent non-tumor tissue samples) from eligible gastric cancer patients. NKX2.1 mRNA levels were significantly reduced in 23 (74.2%) tumor tissue samples relative to the adjacent non-tumor tissue samples (P<0.001, Figure 1).

### NKX2.1 expression analyzed by Western blotting

**Figure 2:**
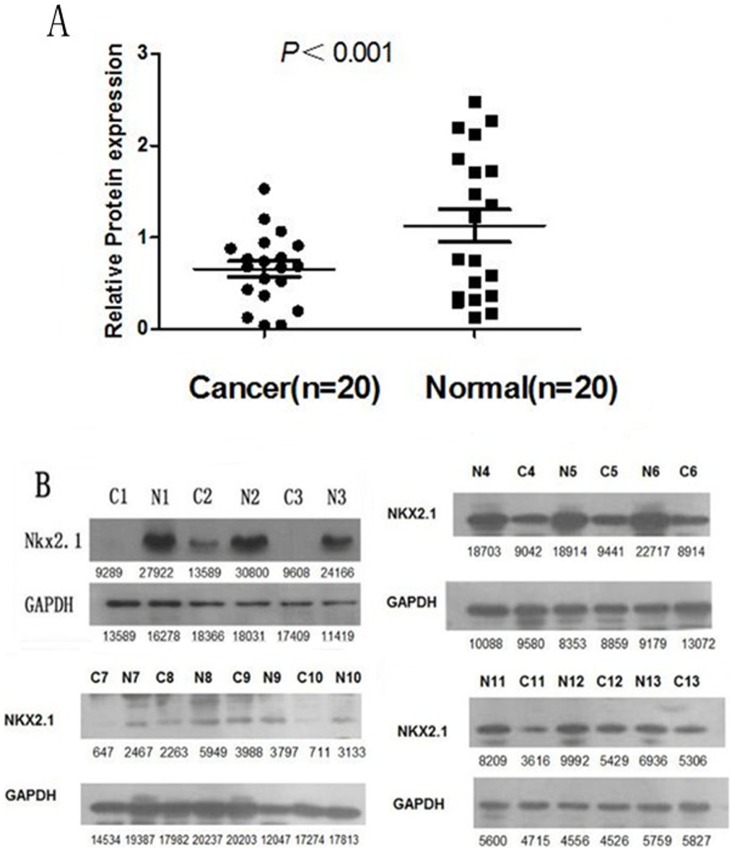
Western blotting analysis of NKX2.1 protein expression in gastric cancer patients. (**A**). NKX2.1 protein expression was decreased in 13 tumor tissues compared with adjacent non-tumorous tissues (p<0.001). (**B**). The NKX2.1 protein expression was lower in the cancer tissues than in matched adjacent non-tumorous tissues.

NKX2.1 protein levels in the resected gastric cancer samples were determined by Western blotting. Our results showed an NKX2.1 band at the expected size of 38 kDa, and the amount of NKX2.1 protein present was then measured by densitometry. As shown in Figure 2, when compared with the matched adjacent non-tumor tissue samples, a decrease in NKX2.1 expression was detected in 13 (65.0%) of the 20 tumor tissue samples (p<0.001, Figure 2A and Figure 2B). These findings were consistent with those from the qRT-PCR analysis.

### The association between the levels of NKX2.1 expression and clinicopathological characteristics, based on immunohistochemical staining

**Figure 3:**
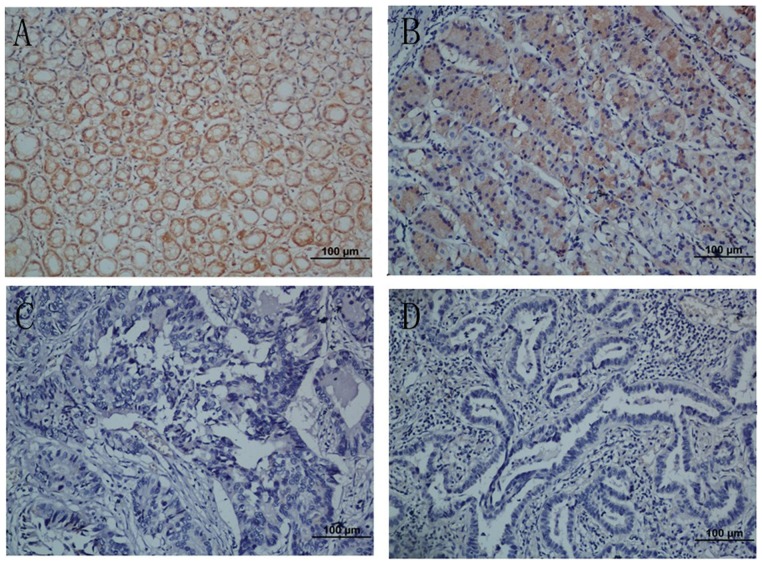
Immunohistochemical analyses of NKX2.1 expression in resection specimens of primary gastric carcinoma. (**A**) Strong NKX2.1 staining was observed in noncancerous gastric mucosa glands. (**B**) Immunostaining of the well-differentiated gastric cancer cells. (**C**) Weak NKX2.1 staining in poorly-differentiated gastric adenocarcinoma. (**D**) NKX2.1-negative gastric adenocarcinoma. Original magnification for A–D, ×200.

To obtain further insight into the effect and prognostic value of NKX2.1 expression in gastric cancer patients, paraffin-embedded tissue sections (n = 205) of histopathologically confirmed gastric carcinoma were examined using immunohistochemistry. NKX2.1 immuno-positivity was significantly different between the tumor tissue samples and the adjacent non-tumor ones. NKX2.1 expression was localized to the cytoplasm in 118 (57.6%) of the tumor tissue samples, whereas the remaining 87 cases (42.4%) displayed low or no cytoplasmic NKX2.1 expression (Table 1). Based on the categories defined above, the expression status of NKX2.1 correlated significantly with age (P = 0.002), tumor size (P = 0.004), depth of tumor infiltration (T stage, P <0.001) and TNM stage (P <0.001) but not with gender (P = 0.861), local lymph node metastasis (N stage, P = 0.325) or distant metastases (M stage, P = 0.612). Representative photomicrographs are shown in Figure 3.

**Table 1:** Correlation between NKX2.1 expression and clinicopathological variables of 205 gastric cancer cases.

Clinicopathological parameters	*n* a	NKX2.1 Expression		χ^2^	*P* Value
		Low	High		
**All**	205	87	118		
**Age (years)**					
<55	84	25	59	9.363	0.002*
≥55	121	62	59		
**Gender**				0.031	0.861
Male	74	32	42		
Female	131	55	76		
**Tumor size**				8.088	0.004*
<3 cm	40	9	31		
≥3 cm	165	78	87		
**Tumor infiltration**				24.503	<0.001*
T1	32	5	27		
T2	22	3	19		
T3	16	9	7		
T4a	104	51	53		
T4b	31	19	12		
**Local lymph node metastasis**				3.468	0.325
N0	66	24	42		
N1	38	19	19		
N2	39	14	25		
N3	62	30	32		
**Distant metastasis**				0.257	0.612
M0	184	77	107		
M1	21	10	11		
**TNM staging**				21.330	<0.001*
I	42	5	37		
II	38	22	16		
III	104	50	54		
IV	21	10	11		

a Numbers of cases in each group.

*Statistically significant (*P*<0.05).

### Expression of NKX2.1 and clinical outcome

**Figure 4:**
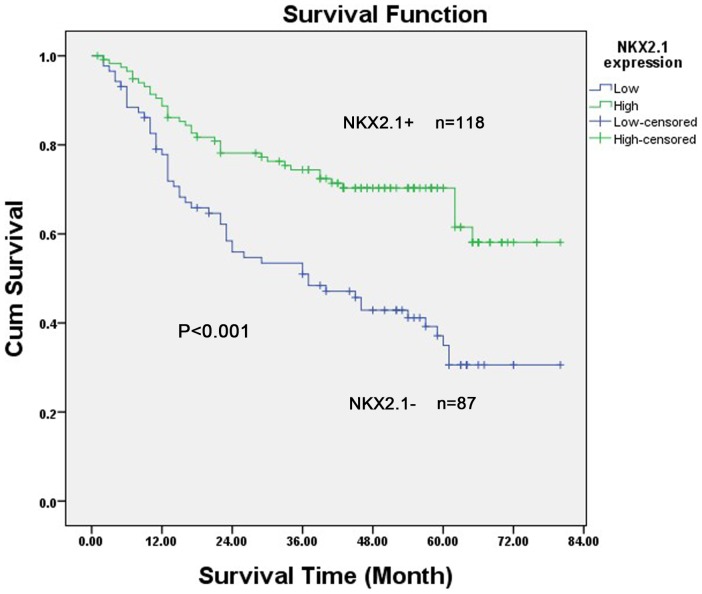
Kaplan–Meier survival curves for subjects with gastric carcinoma (n = 205) patients with high NKX2.1 expression (n = 118) and low NKX2.1 expression (n  =  87) after resection. Patients in the high-expression group exhibited significantly better survival than those in the low-expression group (log-rank test: P<0.001).

The 5-year overall survival rates in patients with high and low NKX2.1 expression were 67.5% and 44.3%, respectively. The overall survival of patients with low NKX2.1 expression was significantly shorter than that of patients with high NKX2.1 expression (P<0.001, log-rank test, Figure 4). Univariate Cox regression analyses showed that the depth of tumor infiltration, local lymph node metastasis, distant metastasis, TNM stage, tumor size and NKX2.1 expression were significantly interrelated with overall survival (Table 2). Furthermore, a multivariate Cox regression analysis confirmed that local lymph node metastasis (P = 0.002), distant metastasis (P<0.001) and NKX2.1 expression (P = 0.005) could serve as independent predictors of the overall survival of patients with gastric carcinoma (Table 2).

**Table 2:** Univariate and multivariate analyses of overall survival of gastric cancer patients.

Variables	*n* a	Univariate analyses			Multivariate analyses		
		HR	(95% CI)	*P* Value	HR	(95% CI)	*P* Value
**Age (years)**				0.067			
<55	84	1.000					
≥55	121	1.489	0.969–2.289				
**Gender**				0.070			
Female	74	1.000					
Male	131	1.499	0.964–2.329				
**Tumor size**				0.001*			0.354
<3 cm	40	1.000			1.000		
≥3 cm	165	5.935	2.176–16.183		1.632	0.580–4.593	
**Tumor infiltration**				<0.001*			0.077
T1	32	1.000			1.000		
T2	22	<0.001	0.000-1E+168		<0.001	0.000-9E+144	
T3	16	0.086	0.026–0.284		0.275	0.079–0.957	
T4a	104	0.363	0.163–0.806		0.414	0.181–0.945	
T4b	31	0.455	0.283–0.370		0.582	0.353–0.961	
**Local lymph node metastasis**				<0.001*			0.002*
N0	66	1.000			1.000		
N1	38	0.114	0.057–0.227		0.280	0.132–0.596	
N2	39	0.399	0.228–0.699		0.534	0.303–0.940	
N3	62	0.396	0.228–0.686		0.548	0.311–0.964	
**Distant metastasis**				<0.001*			<0.001*
M0	184	1.000			1.000		
M1	21	5.701	3.395–9.570		2.523	1.027–6.201	
**TNM staging**				<0.001*			
I	42	1.000					
II	38	0.122	0.000–5.29E109				
III	104	0.292	0.650–0.228				
IV	21	0.343	0.172–0.495				
**NKX2.1**				<0.001*			0.005*
Low	87	1.000			1.000		
High	118	0.425	0.279–0.647		0.518	0.328–0.818	

HR, hazard ratio; CI, confidence interval;

a Numbers of cases in each group; * Statistically significant (*P* < 0.05).

### The role of NKX2.1 in cell proliferation, migration and invasion

**Figure 5:**
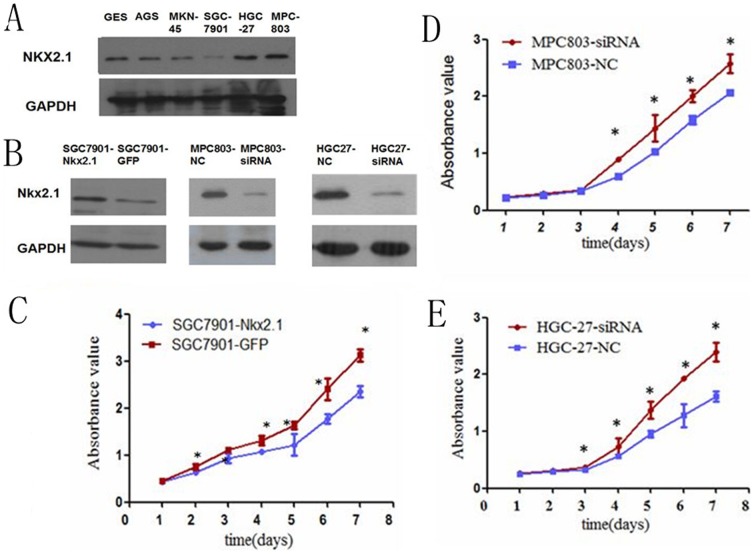
The NKX2.1 protein levels were significantly lower, (A), in the SGC-7901 and higher in the MPC-803 and HGC-27 cell lines than in the normal gastric cell line GES. (**B**) The NKX2.1 expression was significantly higher in the NKX2.1 transfected SGC-7901 cells and lower in the siNKX2.1 transfected MPC-803/HGC-27 cells than controls. (**C-E**) The MTS assay showed that NKX2.1 suppressed the proliferation of the over-expressed SGC-7901 and accelerated the proliferation of the down-regulated MPC-803/HGC-27 cells, * P < 0.05.

**Figure 6:**
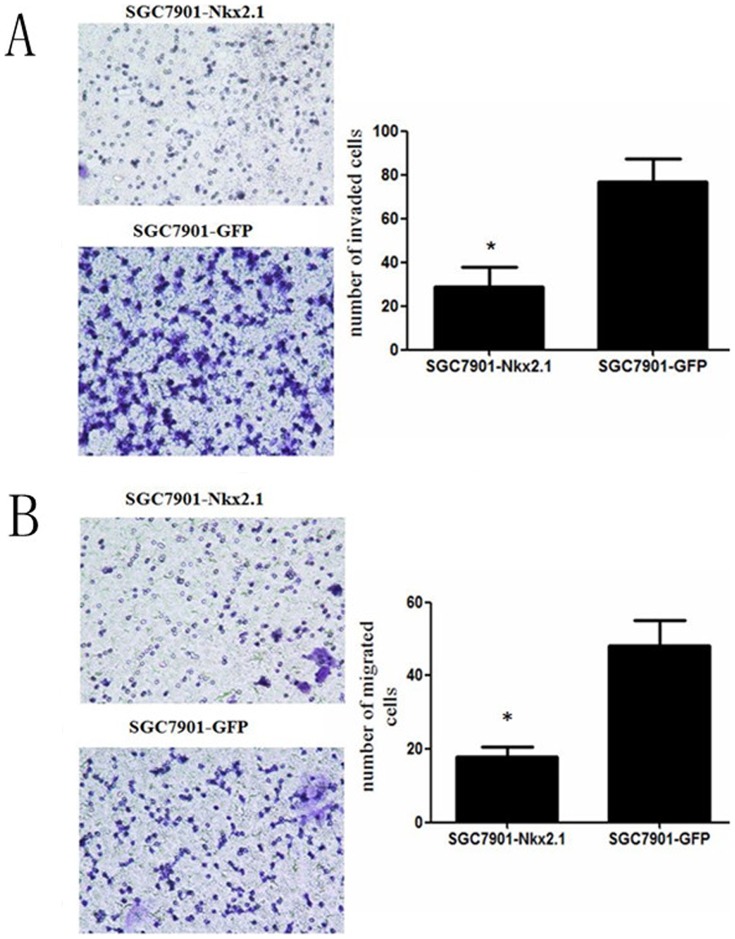
Transwell invasion (A) and migration (B) assays of NKX2.1 over-expressed SGC-7901 cells. Over-expressing NKX2.1 significantly inhibited cell invasion and migration by the SGC-7901 cells, * P < 0.05.

To evaluate the effect of NKX2.1 on cell proliferation a NKX2.1 expression vector and a GFP-control vector were transfected into SGC-7901 cell lines. The efficiencies of transfection were detected by western blotting (Figure 5B). A cell growth assay revealed that cell growth rates in NKX2.1-transfected cell lines were slower than GFP-transfected gastric cancer cell lines (Figure 5C). The overexpression of NKX2.1 dramatically reduced the migration and invasion ability of the cells (Figure 6).

**Figure 7:**
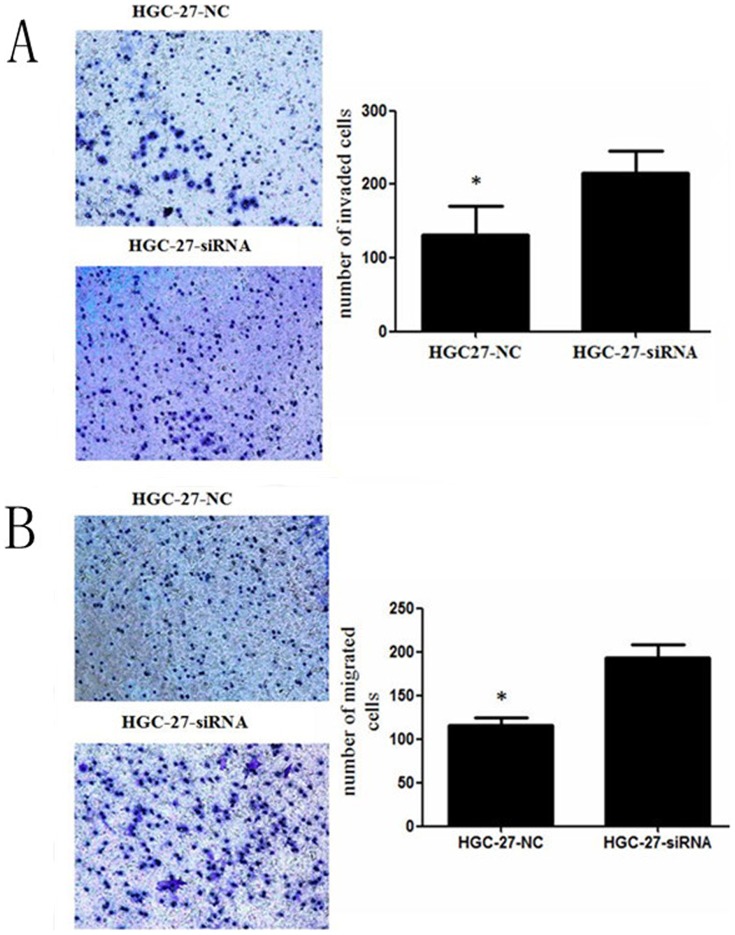
Transwell invasion (A) and migration (B) assays of NKX2.1 down-regulated HGC-27 cells. Silencing NKX2.1 significantly promoted cell invasion and migration by the HGC-27 cells, * P < 0.05.

**Figure 8:**
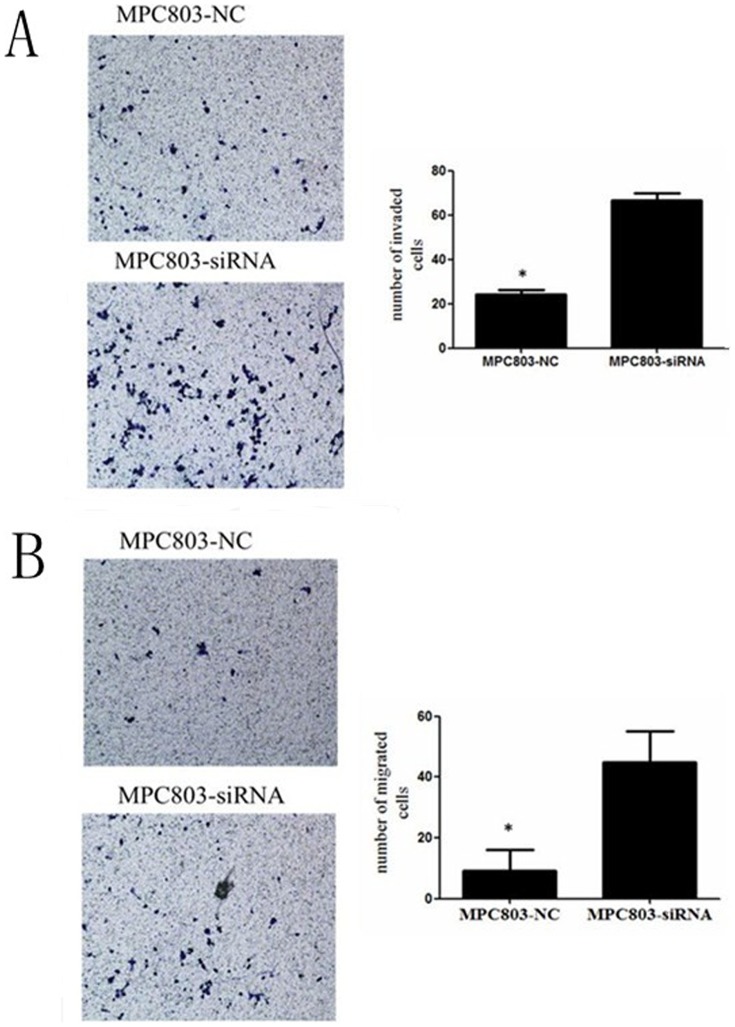
Transwell invasion (A) and migration (B) assays of NKX2.1 down-regulated MPC-803 cells. Silencing NKX2.1 significantly promoted cell invasion and migration by the MPC-803 cells, * P < 0.05.

The expression of NKX2.1 in MPC-803/HGC-27-siNKX2.1cells and MPC-803/HGC-27-NC cells were detected by western blotting (Figure 5B). A cell growth assay and transwell migration and invasion assays displayed that silencing of NKX2.1 accelerated the proliferation (Figure 5D, E) and promoted the migration and invasion of MPC-803 and HGC-27 cells (Figure 7, 8).

## Discussion

Tumor progression arises as a consequence of a series of cellular events that involve but are not limited to the activation of oncogenes and the inactivation of tumor suppressor genes [18]. In the early 1980s, researchers cloned tumor suppressor genes and demonstrated their pivotal role in regulating the differentiation of normal cells [19] . More recently, inactivating mutations of tumor suppressor genes have been frequently detected in human tumor tissues, suggesting that the expression levels of certain tumor suppressor genes could serve as important prognostic factors for the corresponding human cancers.

NKX2.1, also named thyroid transcription factor-1 (TTF-1), was first discovered in normal thyroid tissue [8]. It functions as a tissue-specific transcription factor, regulating the expression of downstream genes and participating in the differentiation and development of normal tissues [20]. Recent studies have shown reduced expression of NKX2.1 in several types of cancerous human tissue, including lung cancer, breast cancer, and colon cancer, demonstrating that NKX2.1 is a putative new tumor suppressor gene in human cancers. Moreover, Anagnostou VK et al. compared the five-year survival rate of non-small cell lung cancer (NSCLC) patients with high or low NKX2.1 expression levels. They found that patients showing high levels of NKX2.1 had a significantly better prognosis [10]. Similar studies have been performed for thyroid cancer and gynecological malignancies, and similar results were obtained for papillary thyroid carcinoma and human ovarian cancer [14]–[17]. These data suggest that NKX2.1 can be utilized as an independent prognostic factor in human cancers.

In our study, the expression of NKX2.1 in primary gastric carcinoma was determined by quantitative real-time PCR (qRT-PCR) and Western blotting. Our qRT-PCR results showed that the expression of NKX2.1 mRNA was reduced in tumor tissue samples compared with that in matched adjacent non-tumor tissue samples (P<0.001); this finding was confirmed by Western blot analysis (P<0.001). Thus, it is possible for an inactivating mutation of the NKX2.1 gene to arise in gastric cancer tissues and serve as an initiating event in the tumorigenesis of gastric cancer. Based on immunohistochemical staining data, we hoped to demonstrate the prognostic value of NKX2.1 for gastric cancer patients. NKX2.1 expression was significantly decreased in 87 of 205 (42.4%) gastric carcinoma cases. A Kaplan-Meier survival analysis showed a significant correlation between the reduced expression of NKX2.1 and poorer clinical outcome of gastric cancer patients after radical operation (P<0.001). Moreover, univariate and multivariate Cox regression analyses further demonstrated that lower NKX2.1 expression is an independent risk factor for overall survival. In our *in vitro* study, NKX2.1 over-expression diminished the proliferation, migration, invasion of gastric cancer cell lines. Moreover, siNKX2.1 transfected cells showed stronger ability of proliferation, migration and invasion. Consistent with the idea proposed in previous studies that NKX2.1 may function as a prognostic factor for some types of cancer, our conclusion supports the hypothesis that NKX2.1 can serve as an independent prognostic factor for gastric cancer patients after surgery. These data suggest that the examination of NKX2.1 expression might be helpful in guiding clinical management for GC.

As an upstream regulatory gene, the function of NKX2.1 depends on perplexing downstream pathways. Recently, researchers have begun to shed light on the molecular function of NKX2.1 in tumorigenesis. In 2011, Winslow MM et al. published an article in the journal *Nature*, in which they investigated the probable mechanism of NKX2.1 as a tumor suppressor. After studying NKX2.1 gene function in a mouse model of lung carcinoma, the researchers proposed that NKX2.1 might regulate the differentiation of tumor cells and thus limit their metastatic potential [11] . Recent reports clarified that the reduced expression of NKX2.1 is associated with the dedifferentiation of tumor cells and an increase in their metastatic capacity. However, earlier studies found NKX2.1 to be overexpressed in human lung carcinoma, so NKX2.1 was once surmised to be an oncogene [20], [21]. Whether NKX2.1 is an oncogene or tumor suppressor remains a hot debate in the field of cancer research. Experts who support the tumor suppressor gene hypothesis demonstrated that NKX2.1 could decrease the expression of occludin, which is a group of proteins localized to and regulating intercellular junctions, thus inhibiting the metastasis of malignant cells [22]. Another hypothesis proposes that NKX2.1 negatively regulates the expression of myosin binding protein H and increases the migratory ability of tumor cells, thereby functioning as a cancer-promoting gene [12]. Therefore, further investigation into the molecular mechanism of NKX2.1 is anticipated.

In summary, our data suggest that NKX2.1 may function as a tumor suppressor in primary gastric carcinoma and that its reduced expression independently predicts an unsatisfactory prognosis in gastric carcinoma patients. We anticipate that NKX2.1 will serve as a valuable prognostic marker in the future.
